# High Rate of Transplantation Before Review of Status Exception Requests Among Adult Heart Transplant Candidates

**DOI:** 10.1161/CIRCHEARTFAILURE.125.013994

**Published:** 2026-06-22

**Authors:** Daniel J. Ahn, Toshihiro Nakayama, Antony Attia, Molly White, Dalin Eap, Nikhil Narang, Kiran K. Khush, William F. Parker, Kazunari Sasaki

**Affiliations:** Department of Surgery (D.J.A., T.N., A.A., K.S.), Stanford University, CA.; Department of Medicine (K.K.K.), Stanford University, CA.; Stanford Cardiovascular Institute (D.J.A., K.K.K.), Stanford University, CA.; Stanford Transplant Outcomes Research Center, Stanford University, CA (D.J.A., T.N., A.A., K.S.).; Department of Medicine (M.W., N.N., W.F.P.), University of Chicago, IL.; Department of Public Health Sciences (W.F.P.), University of Chicago, IL.; Doctoral Diversity Program, Johns Hopkins University, Baltimore, MD (D.E.).; MacLean Center for Clinical Medical Ethics, University of Chicago, IL (W.F.P.).

**Keywords:** heart transplantation, medical ethics, transplant recipient

## Abstract

**BACKGROUND:**

In the US heart allocation system, when transplant centers submit applications for status exceptions to increase waitlist priority, patients obtain the requested status upgrades immediately while their applications are sent to the regional review boards (RRBs) and reviewed retrospectively. How often transplants occur during this period is unknown.

**METHODS:**

Using the Scientific Registry of Transplant Recipients, we identified all adult heart transplant candidates listed between October 18, 2018, and May 31, 2025, with submitted applications for status exceptions. We assessed (1) the time elapsed between submission of exception applications and their receipt by the RRBs and (2) the rate of heart transplantation during this travel time, stratified by whether the applications were eventually approved or denied. Additionally, we estimated how many listed patients were skipped by candidates who received transplants with exceptions that were ultimately denied.

**RESULTS:**

138 transplant centers submitted status exception requests on behalf of 11 508 adult candidates during the study period, of whom 913 (7.9%) received a denial at least once. The median time from obtaining status upgrades to application receipt by the RRBs was 3 days. Three thousand seven out of 11 508 (26.1%) patients received transplants before the RRBs even received their applications, with 174 (19.1%) among 913 with eventual denials and 2833 (26.7%) among 10 595 with approvals. The cumulative incidence of heart transplantation before application receipt for eventual denials was 19.1% (95% CI, 16.6%–21.7%), and that for approvals was 27.2% (95% CI, 26.4%–28.0%; *P*<0.001) at 2 weeks. Candidates who received transplants despite being denied exceptions bypassed more than 11 thousand potential transplant recipients.

**CONCLUSIONS:**

More than 25% of patients with status exception requests receive heart transplants before their applications are even received by their RRBs, raising significant concerns about the fairness of retrospective review of exception requests for the allocation of donor hearts.

**What is New?**

- This study is the first to quantify when adult heart transplants occur relative to the review of status exception requests.
- Between October 2018 and May 2025, >25% of patients with exception requests receive transplants before the regional review boards even receive their review applications.
- 174 patients obtained transplants with status exception requests that were ultimately denied by the regional review boards, bypassing thousands of potential transplant recipients.

**What Are the Clinical Implications?**

- The retrospective review system of exception requests provides insufficient oversight, allowing numerous patients to receive heart transplants with elevated priority before the regional review boards can determine whether the requests meet approval standards.
- This framework undermines fair allocation and permits transplantation of patients whose medical urgency does not justify their waitlist priority.
- Prospective review of exception requests is important for ensuring that donor hearts are allocated to patients with the highest medical urgency.

**See Editorial by Aleksova**

Since October 2018, the US donor heart allocation system has rank-ordered adult transplant candidates using 6 ordinal statuses. To qualify for the highest priority statuses, most patients must have objective hemodynamic or laboratory evidence of cardiogenic shock.^1^ However, the system allows transplant centers to request exceptions on behalf of select candidates. In exception applications, centers may argue to their respective regional review board (RRB) that their patients have a similar level of medical urgency as others who have qualified for the same status by meeting standard criteria. One of the goals of the 2018 heart allocation policy change was to reduce the system’s dependence on exception listings by increasing the number of available statuses.^2^ However, the proportion of listings with exceptions has increased dramatically to about 30% to 40%.^3–7^ Additionally, the approval rate for heart exception requests is consistently >95%, with most denials eventually getting approved through appeals.^3^ Worryingly, candidates listed with status exceptions have significantly reduced waitlist mortality compared with those who are listed via standard criteria.^8,9^

As the heart transplant community seeks to limit the overuse of exceptions, 1 aspect of exception allocation that has not been previously studied is how exception requests are reviewed retrospectively. On submission of exception requests, all applicants immediately receive the requested status and accrue high-priority listing time while the requests are still in transit to the RRBs and are either approved or denied retrospectively through a voting process that may take up to a week.^10^ If denied, transplant centers may submit appeals to the RRB, and if still unfavorable, to the Organ Procurement and Transplantation Network (OPTN) Heart Committee, all the while patients continuing to possess the requested status.^1^ In this study, we determined how much time candidates spend with status upgrades before the RRBs receive their exception applications and how often heart transplants occur during this period.

## Methods

### Data Source and Study Population

This study used data from the Scientific Registry of Transplant Recipients (SRTR). The SRTR includes data on all donors, waitlisted patients, and transplant recipients in the United States, submitted by the members of the OPTN. The Health Resources and Services Administration provides oversight to the activities of the OPTN and SRTR contractors. The files used include the standard analysis files, the heart status justification files, and complete match run data. This study was exempted by the University of Chicago and Stanford University Institutional Review Boards. We followed STROBE/RECORD guidelines in conducting this study. Access to the data requires completion of a data use agreement and approval from the SRTR. Complete statistical code necessary to reproduce the study results is available in a repository online.^11^

We identified all adult heart transplant patients in the SRTR data set listed between October 18, 2018, and May 31, 2025, who received a status exception at least once during listing, with follow-up until June 30, 2025. We collected relevant demographic and clinical data from patients at initial listing. Variables included sex, age at listing, body mass index, race, blood type, primary diagnosis, and mechanical circulatory support devices at initial listing, cardiac index at initial listing, and pulmonary capillary wedge pressure at initial listing. We categorized body mass index into the following: underweight (<18.5 kg/m^2^), normal (18.5–24.9 kg/m^2^), overweight (25–29.9 kg/m^2^), and obese (≥30 kg/m^2^). We also calculated the medical urgency of all candidates at initial listing with the United States Candidate Risk Score (US-CRS), a new continuous medical urgency score that is more accurate in predicting waitlist mortality of adult heart transplant candidates than the 6-status system. The US-CRS is a linear combination of 5 laboratory values (bilirubin, sodium, eGFR, albumin, and BNP/NT-proBNP [N-terminal pro-B-type natriuretic peptide]), an indicator for durable left ventricular support device support, and an indicator for venoarterial extracorporeal membrane oxygenation (VA-ECMO) and temporary surgical VAD. Values range from 1 to 50, with larger numbers indicating greater medical urgency.

### Outcomes and Analysis

**Figure 1.**
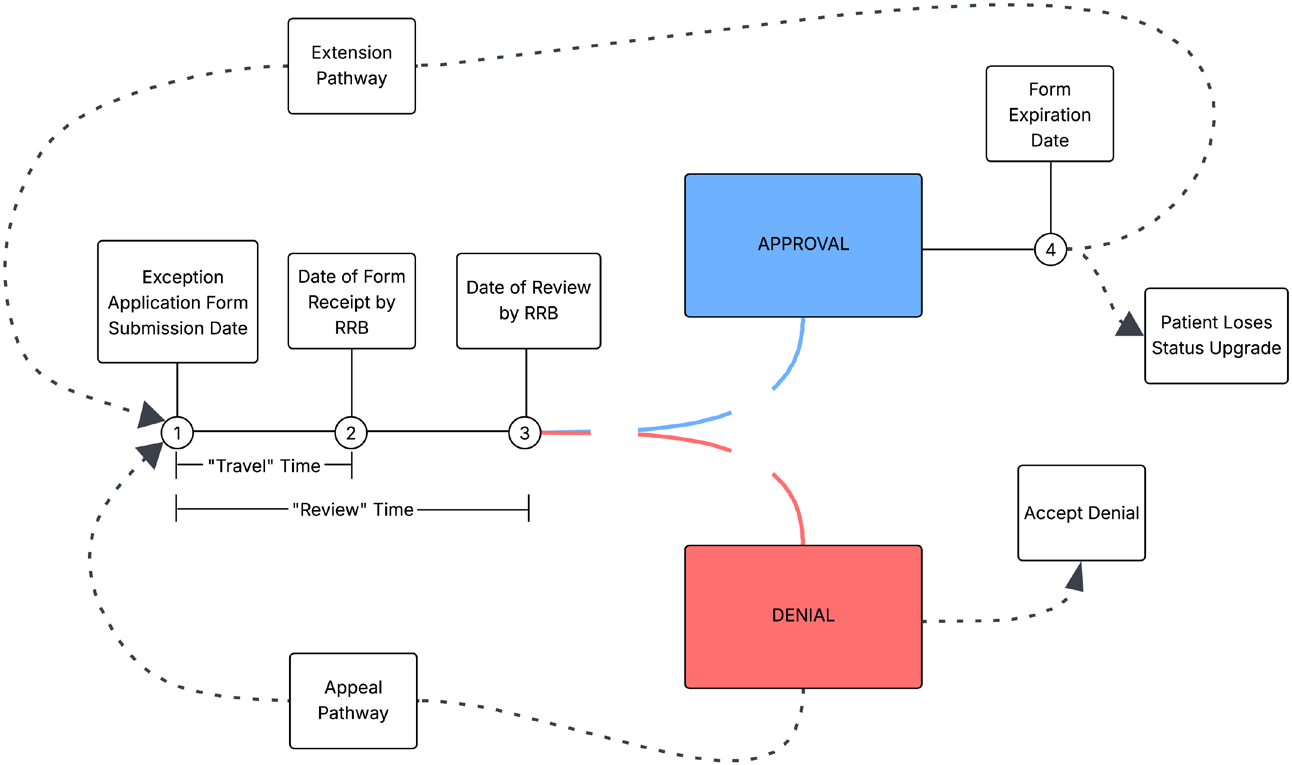
**Graphical representation of the retrospective review process of applications for status exceptions in the adult heart allocation system.** Listing centers may submit initial applications for status exceptions at Timepoint 1, and patients obtain the requested statuses immediately. After a travel time, the regional review boards (RRBs) receive the applications at Timepoint 2 and make a final decision through a majority voting process at Timepoint 3, which completes the review time. If approved, patients keep the requested status they obtained at Timepoint 1 until their status upgrade expires at Timepoint 4, which is based on the status (eg, 7 days for status 1). Before Timepoint 4, centers may either allow the approved forms to expire and have patients lose the upgraded status or submit applications to extend the exceptions, which restarts the review process at Timepoint 1. If initial applications are denied at Timepoint 3, centers may accept the denial, and patients drop to the status they meet through standard criteria. Alternatively, centers may submit forms for appeal within a required amount of time, which causes the review process to start again at Timepoint 1. Because review of exception applications is retrospective, patients always have the requested status throughout the review time, even if their applications are denied at Timepoint 3. Furthermore, while undergoing the appeal pathway, candidates possess the same requested status they had since submission at Timepoint 1, even with denial by the RRBs.

The primary outcome of the study was heart transplantation. The secondary outcome was the time elapsed between patients obtaining status exceptions and the RRBs receiving the respective exception applications for review. Because the date of final approval or denial by the RRB is not included in the files provided by the SRTR, we were unable to determine the time elapsed between exception application submission and final review by the RRB. A graphical representation of the exception review process is in Figure 1. More information on how we determined when the RRBs received the exception application forms is in the Supplemental Methods (section A, Table S1).

We first identified all initial exception applications (for initial listings and status extensions) that were submitted and excluded any requests that were never reviewed by the RRBs. We then calculated the approval rate, stratified by United Network for Organ Sharing region. We also calculated the proportion of all waitlisted candidates who submitted exception applications, stratified by transplant center. Next, we calculated the amount of time that elapsed between patients obtaining status designations through exceptions and the receipt of the exception applications by the respective RRBs. We stratified this travel time by both requested status on the waitlist and United Network for Organ Sharing region. We compared the median travel time by region for each status with the Kruskal-Wallis test.

Furthermore, we identified all heart transplant candidates who received a heart transplant while their exception applications were still en route to the RRBs, stratified by whether they were eventually approved or denied. To identify whether there was a trend in the absolute number of transplants occurring before form receipt by the RRBs over time, we performed the Mann-Kendall test.^12^ The test statistic τ ranges from −1 to 1, with a positive τ indicating an upward trend and a negative τ a downward trend; values closer to 1 or -1 suggest stronger trends.

For the patients who received transplants with exceptions that were eventually denied, we estimated what status they would have had at the time of transplant without the exceptions. To perform this analysis, for patients who were already on the waitlist, we simply carried forward the status they had before submitting a status exception. For those who were inactive immediately before obtaining an exception or who received an exception at the time of initial listing, we determined what status they would have had based on OPTN heart policy criteria using information on therapies such as mechanical circulatory support and hemodynamic measurements documented in the status justification files. More information on how we determined what patients’ statuses would have been without exceptions is in the Supplemental Methods (section B).

We next utilized complete match run data provided by the OPTN to estimate how many potential transplant recipients were skipped due to candidates getting listed at a higher status by exceptions that were eventually denied. Because match run data up to December 31, 2024, was available for this analysis, we did not perform a match run analysis for the donor hearts that were transplanted in 2025. For simplicity of analysis, we did not calculate waiting time to determine the exact predicted placement in the match run. Instead, we assumed that for given predicted statuses without exceptions, candidates were at the top of their respective sequence classification in the match run. In effect, this provides the most conservative estimate of the true number of skipped potential transplant recipients. For the skipped potential transplant recipients, we then estimated the cumulative incidence of death or removal for clinical deterioration within 6 weeks of the match run, treating transplantation as a competing event. More information on this analysis is available in the Supplemental Methods (section C; Tables S2 through S4).

We also calculated the cumulative incidence of transplant before the RRBs received applications for exception requests within 2 weeks. For this patient-level analysis, each patient contributed 1 observation based on a single exception application. For patients with at least 1 denial, we used their last denied exception application to define time 0. For patients with only approvals, we used their last submitted exception application. This approach ensures that the denied group represents those who received transplants during review of applications that were ultimately rejected by the RRBs. In this analysis, there were 2 competing events: (1) death or waitlist removal for clinical deterioration before application receipt and (2) application receipt by the RRBs. Patients were censored if none of these events occurred. We chose 2 weeks, as this was the longest recorded time from submission to receipt in the data set. We then compared the respective rates, stratified by exception approval or denial, with Fine-Gray analysis.

Because the SRTR lacks data on the date of review, we performed a sensitivity analysis estimating the cumulative incidence of transplant before an estimated review date of 3 days after application receipt. We chose 3 days because after an RRB receives an application, the primary representative assigned to the case must vote within 3 days, after which all RRB members cast votes.^10^ We then calculated how many more heart transplants occurred with this change. Finally, to compare demographic and clinical data of patients by approval or denial of exceptions, we performed descriptive statistics with χ^2^ tests for categorical variables and Wilcoxon rank-sum tests for continuous variables. All statistical tests were 2-sided, and we considered a *P* value of <0.05 to be significant. We performed all analyses with R (version 4.5.1).

## Results

Between October 18, 2018, and May 31, 2025, 30 369 adult patients were added to the heart transplant waitlist at 143 US transplant centers, of whom 11 508 patients at 138 centers had at least 1 status exception request submitted on their behalf. The per-center rate of candidates with submitted exceptions differed significantly by transplant center from 0% to 100%, *P*<0.001 (median, 25.9%; Figure S1). 10 595 patients (92.1%) had all exception applications approved by their RRBs, while 913 (7.9%) submitted at least 1 exception application that was denied by the RRBs (Table). Patients with denials were more likely to be male (77.3% versus 72.8%; *P*=0.003), have obesity (41.8% versus 36.9%; *P*=0.02), have blood type O (56.1% versus 46.6%; *P*<0.001), have requested higher statuses (status 1: 21.2% versus 15.0%; *P*<0.001), and have lower ECMO (1.6% versus 12.1%; *P*<0.001) and higher left ventricular assist device (60.7% versus 46.9%; *P*<0.001) utilization at initial listing. There were no significant differences in age at listing or race. The most common diagnosis was nonischemic dilated cardiomyopathy (37.7%), followed by ischemic cardiomyopathy (25.4%). Compared with candidates with denied exceptions, those with approvals had a significantly higher US-CRS at initial listing (39 versus 36; *P*<0.001).

**Table.**
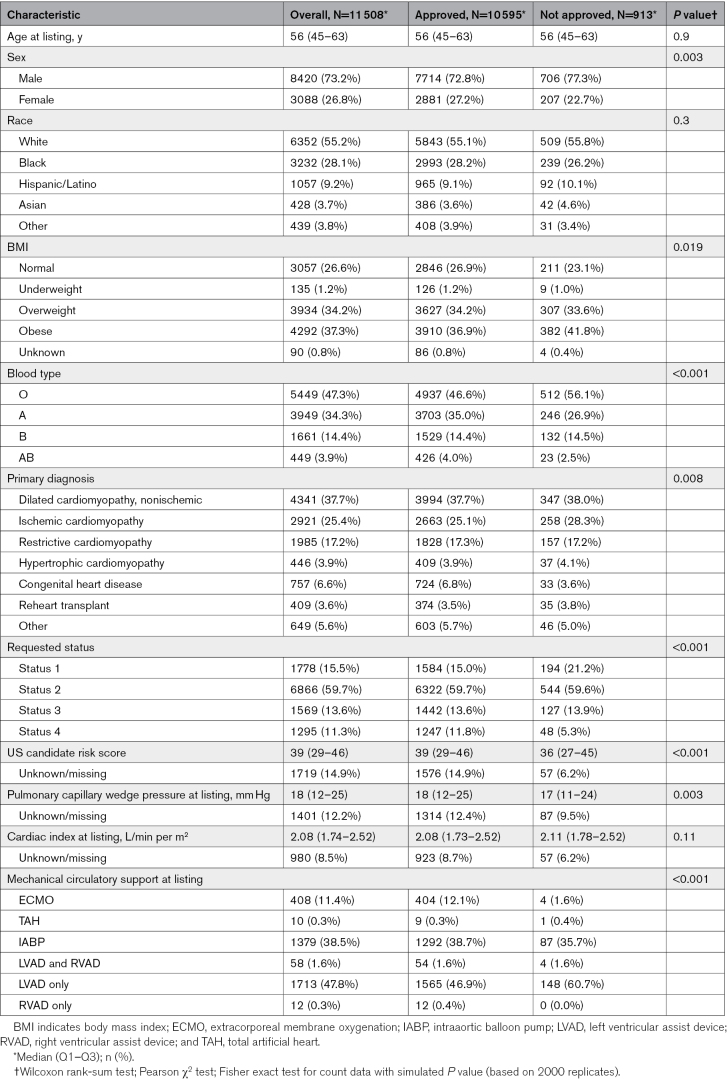
Demographic and Clinical Characteristics of Study Population, Stratified by Approval and Denial of Exception Applications

### Elapsed Time from Use of Exception to Receipt of Exception Application by RRBs

**Figure 2.**
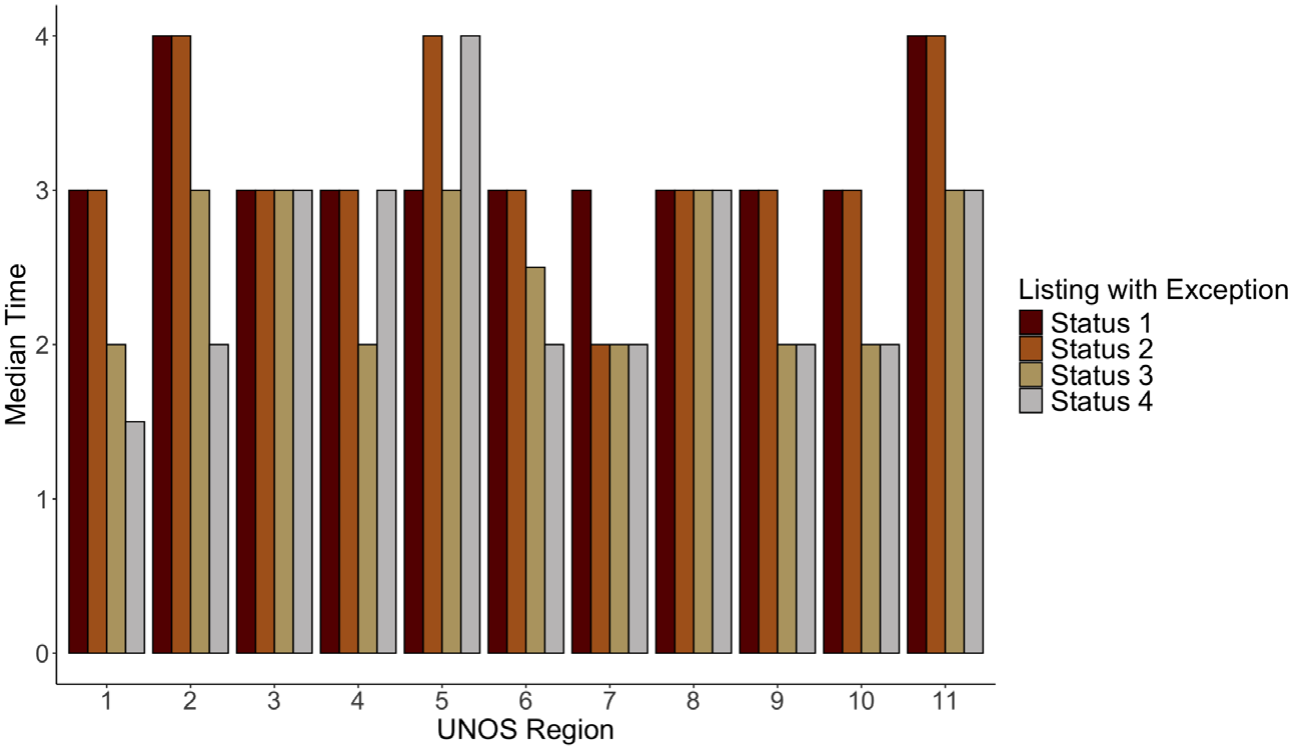
**Median time from exception application submission to application receipt.** Time is stratified by United Network for Organ Sharing (UNOS) region and requested status upgrade. The median travel time overall and by each requested status was 3 days. The travel time varied significantly by UNOS region for all 4 status designations (statuses 1–4; all *P*<0.001).

There were 32 720 exception applications submitted (both requests for initial listing and extensions). After excluding 6 applications because the RRBs never reviewed them, 32 714 requests on behalf of 11 508 candidates were evaluated in this study, with a 96.9% approval rate overall. The approval rate varied significantly by United Network for Organ Sharing region from 94.5% in region 8 to 97.9% in region 4 (*P*<0.001; Figures S2 and S3). The number of applications for statuses 1 to 4 were 2583 (7.9%), 17 245 (52.7%), 8443 (25.8%), and 4443 (13.6%), respectively. The median travel time for all status exception applications to be received by the RRBs was 3 days. The travel time varied significantly by region for all 4 status designations (statuses 1–4; all *P*<0.001; Figure 2).

### Heart Transplantation before Receipt of Exception Applications by RRBs

**Figure 3.**
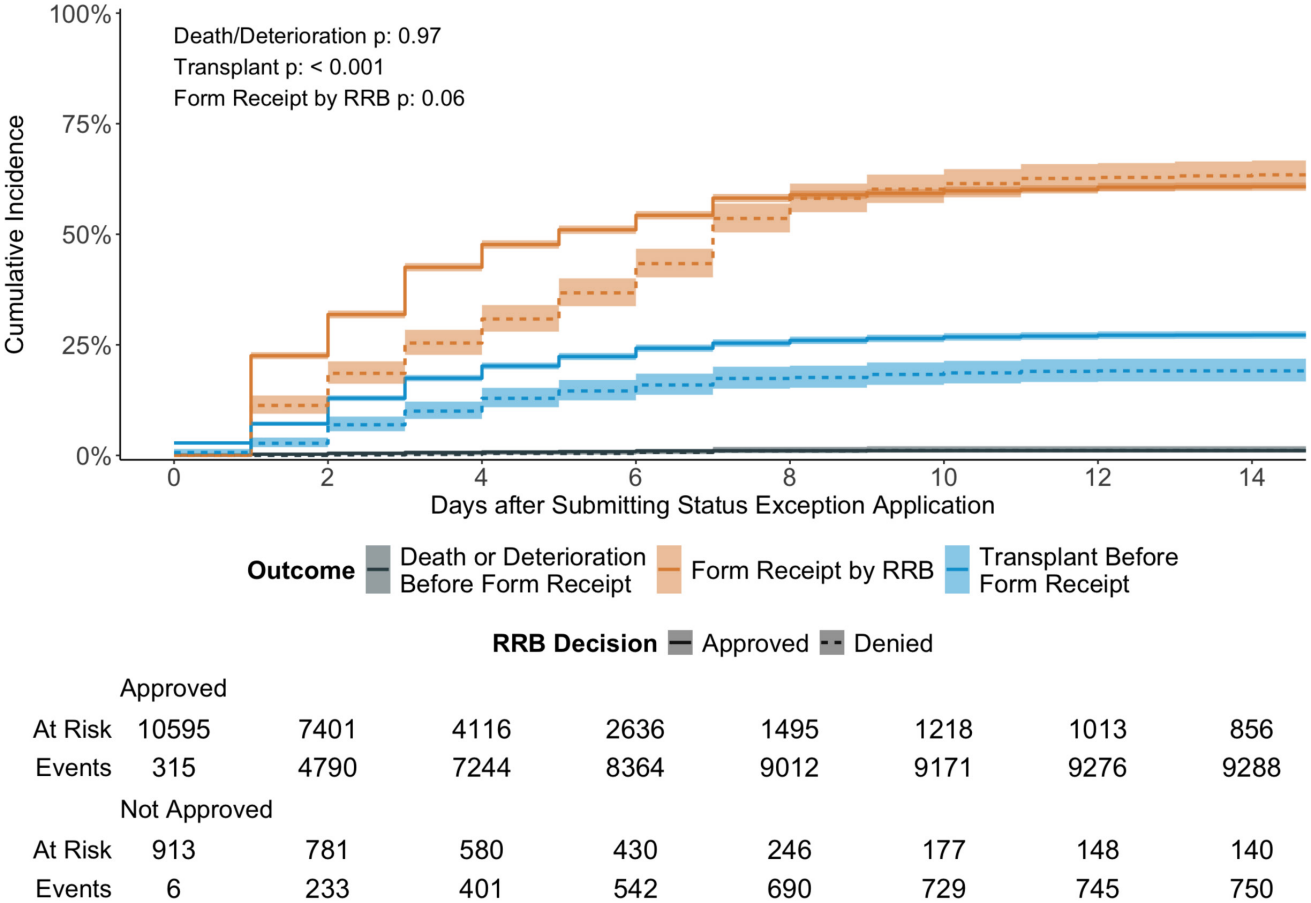
**Heart transplantation before receipt of applications for status exceptions by regional review boards (RRBs).** This shows the cumulative incidence of transplant before application receipt by the RRBs, treating death or waitlist removal for clinical deterioration before application receipt and receipt of applications by the RRBs as competing events. Plots for application approvals are denoted by solid lines, and those for denials are denoted by dotted lines. Fine-Gray analysis results for all competing events stratified by approval or denial of exception applications are in the top-left corner.

**Figure 4.**
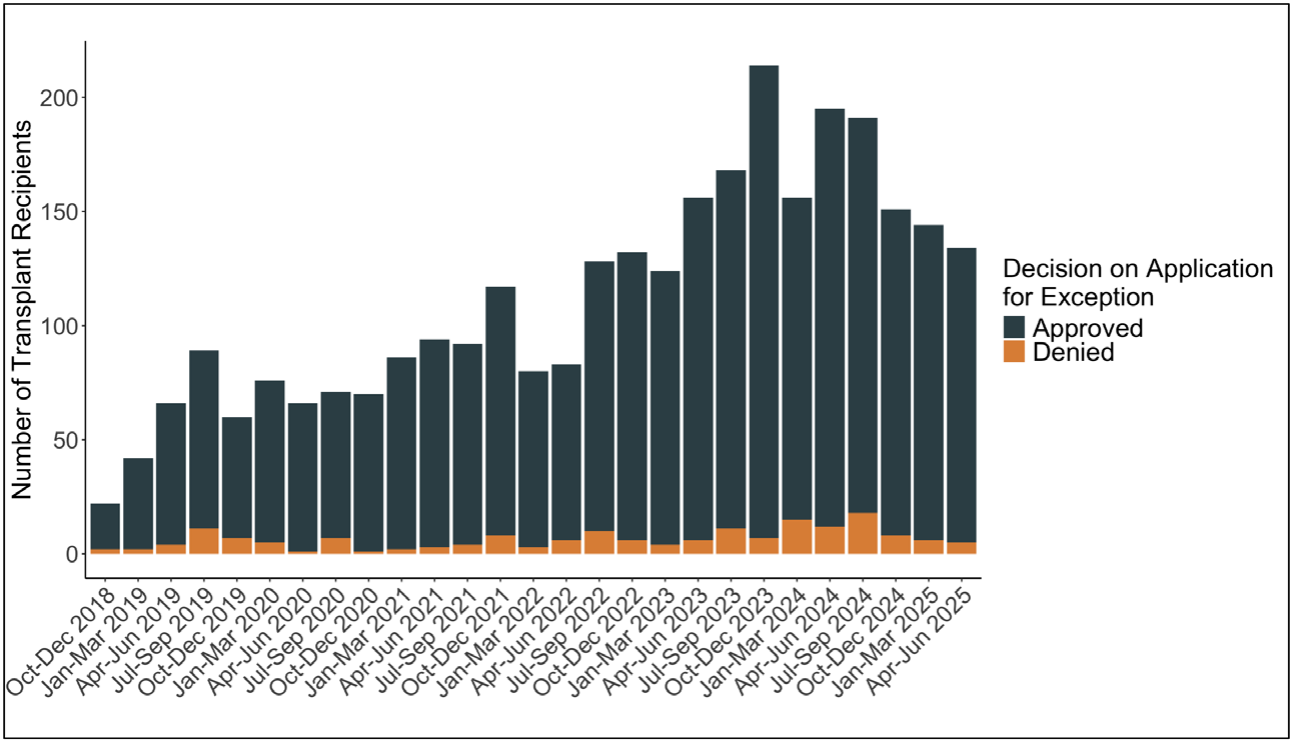
**Trend of heart transplants with exceptions before application receipt by the regional review boards (RRBs).** Black refers to transplants before review by RRBs, with requests for exceptions that were approved, and orange refers to those with requests that were eventually denied. The total number of these transplants has increased since the policy change in October 2018 (τ=0.71; *P*<0.001).

**Figure 5.**
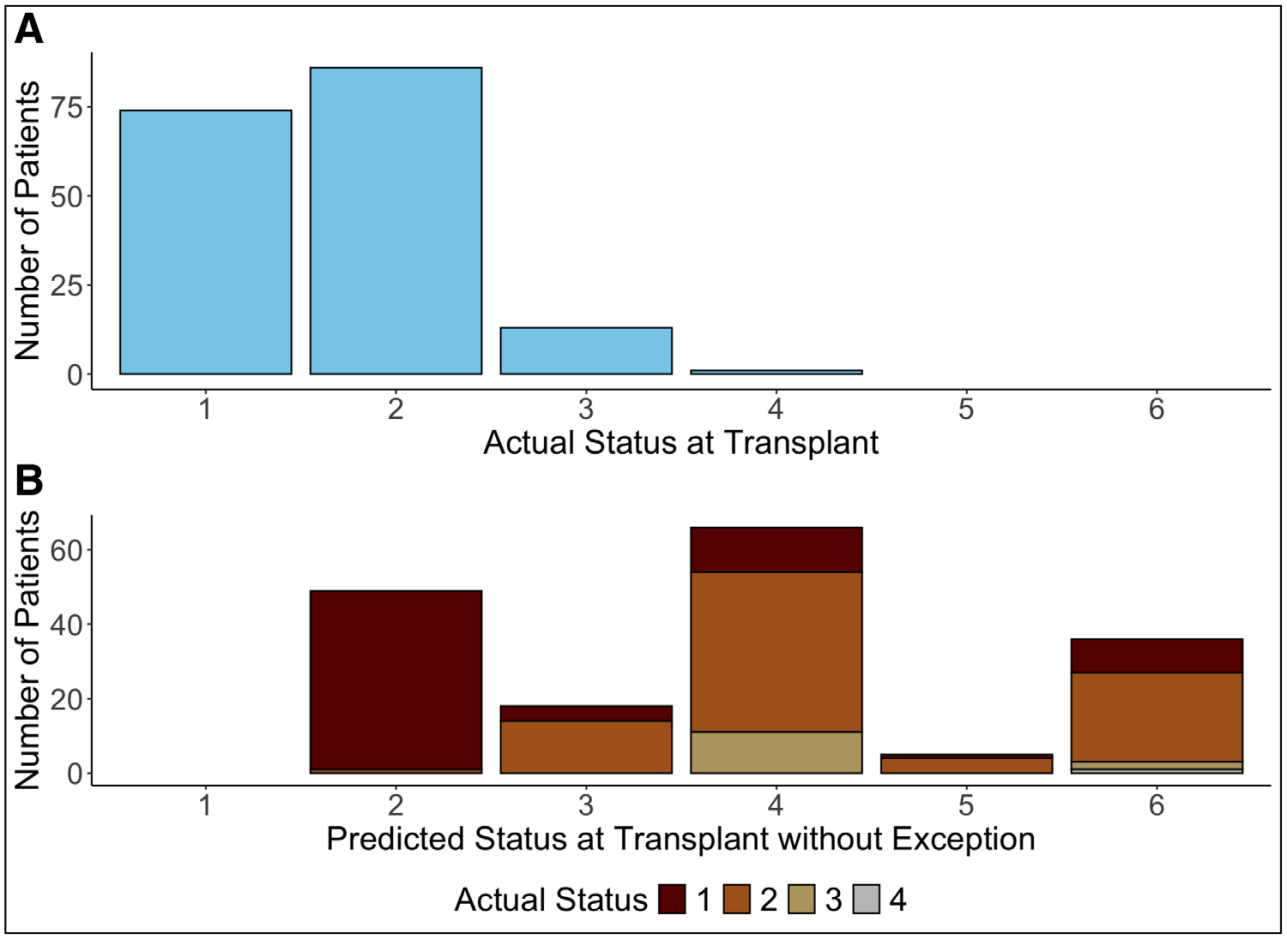
**Distribution of actual and predicted statuses without exceptions at transplant. A**, Actual distribution of status levels at transplant of the 174 patients who received a transplant with exceptions that were ultimately denied by the regional review boards (RRBs). **B**, Distribution of the 174 patients’ predicted statuses without exceptions at the time of transplant based on standard criteria described in Organ Procurement and Transplantation Network (OPTN) policy documents, stratified by what their statuses actually were in 5A. Median United States Candidate Risk Score (US-CRS) at initial listing (available for 148/174 patients, 85.1%) by actual status at transplant: status 1: 43 (interquartile range [IQR] 36–48; n=59), status 2: 40 (IQR, 33–44; n=80), status 3: 43 (IQR, 28–48; n=8), status 4: 47 (n=1).

For the 913 patients with denied exception applications, the cumulative incidence of heart transplantation before application receipt was 19.1% (95% CI, 16.6%–21.7%) at 2 weeks, while that for the 10 595 patients with approvals was 27.2% (95% CI, 26.4%–28.0%; *P*<0.001; Figure 3). The cumulative incidence of death or removal for deterioration for denied patients was 1.1% (95% CI, 0.6%–2.0%) and for approved patients was 1.1% (95% CI, 0.9%–1.3%; *P*=0.97). Three thousand seven out of 11 508 (26.1%) patients received transplants before the RRBs even received their applications. There was a significant increase over time in the number of transplants occurring before form receipt by RRBs (τ=0.71; *P*<0.001; Figure 4). During the travel time, 174 (19.1%) among 913 patients with eventual denials received a transplant (74 with status 1, 86 with status 2, 13 with status 3, and 1 with status 4), and 2833 (26.7%) among 10 595 with eventual approvals were transplanted (Figure 5A). 124 (1.1%) deaths or waitlist removals for clinical deterioration occurred during the travel time. The distribution of the statuses of the 174 patients at the time of transplant is in Figure 5B, stratified by the statuses that they temporarily achieved with their exception requests. 48 (64.9%) out of 74 patients with status 1 would have had status 2. 43 (50.0%) and 24 (27.9%) out of 86 patients transplanted with status 2 exceptions would have had status 4 and 6, respectively.

### Match Run Analysis

Complete match run data up to December 31, 2024 were available for 163 (93.7%) out of 174 patients who received prioritized transplants with exceptions that were ultimately denied. Had they remained in their expected status at the time of their match runs, the 163 donor hearts could have been offered to 11 374 other candidates. Within 6 weeks of the match run dates, 163 (1.4%) of the 11 374 skipped candidates died or were removed from the waitlist for clinical deterioration, and 3898 (34.2%) received heart transplants. The cumulative incidence of death or removal for clinical deterioration within 6 weeks of the match runs was 1.5% (95% CI, 1.3%–1.7%) while that for transplant was 34.9% (95% CI, 34.0%–35.8%]; *P*<0.001; Figure S4).

### Sensitivity Analysis

**Figure 6.**
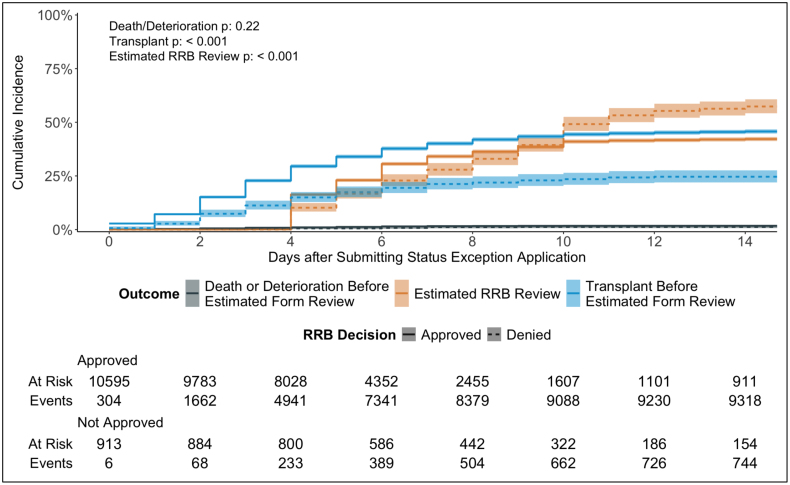
**Heart transplantation before the estimated review of status exceptions by regional review boards (RRBs).** This shows the cumulative incidence of transplant before an estimated review date of 3 days after application receipt by the RRBs, treating death or waitlist removal for clinical deterioration before application receipt and receipt of applications by the RRBs as competing events. Plots for application approvals are denoted by solid lines, and those for denials are denoted by dotted lines. Fine-Gray analysis results for all competing events stratified by approval or denial of exception applications are in the top-left corner.

The number of transplants before an estimated review date of 3 days after application receipt for denied and approved patients was 226 (+52, increased by 29.9%) and 4769 (+1936, increased by 68.3%), respectively. The total of pre-review deaths and removals for deterioration was 196 (+72, increased by 58.1%). The cumulative incidence of transplant before estimated RRB review increased for patients with denials at 24.6% (95% CI, 21.9%–27.5%) and with approvals at 45.7% (95% CI, 44.8%–46.6%) at 2 weeks (*P*<0.001; Figure 6). The cumulative incidence of death or removal for clinical deterioration before estimated review remained low for patients with denials at 1.2% (95% CI, 0.7%–2.1%) and for those with approvals at 1.8% (95% CI, 1.5%–2.0%) at 2 weeks (*P*=0.22).

## Discussion

In this registry-based study of waitlisted patients awaiting heart transplantation with status exceptions in the US, we report the time elapsed between obtaining exceptions and receipt of applications by the RRBs, as well as the rate of heart transplantation while applications are still in transit to the RRBs. Our main findings are as follows:

1. The median time it takes for exception applications to be received by RRBs is 3 days, with significant regional variation for all requested statuses.
2. More than 25% of all patients with exception requests received heart transplants before their applications were even received by their respective RRBs.
3. The number of transplants occurring before exception requests are received by the RRBs has increased significantly over time since October 2018.
4. One hundred seventy-four people received transplants, with the exception of requests that were ultimately denied, bypassing thousands of potential transplant recipients with higher medical urgency, with inappropriately elevated priority.

The total number of transplants that occurred before the receipt of exception applications by the RRBs is likely an underestimate of how many transplants actually occur before final decisions are made. According to OPTN policy,^10^ the primary RRB representative initially assigned to a case has up to 3 days from receipt of an exception application to cast a vote. If the representative is unable to do so, the case is sent to an alternate representative, who then has an additional 4 days. Subsequently, all RRB representatives cast votes, with final approval or denial decided by the majority. In the event of tiebreakers, the RRB chair must cast a vote, which may require additional time. Indeed, in our sensitivity analysis, by estimating the review date to be 3 days after application receipt, transplants increased by nearly 30% for patients with denials and nearly 70% for those with approvals. While there was a significant difference in the cumulative incidence of transplant between approved and denied patients, this was most likely due to the difference in size of the groups, as denied candidates accounted for <8% of the study population.

The fact that more than 3000 candidates have received transplants with status upgrades before the RRBs even reviewed their exception requests since October 2018 is concerning. For these transplants, the exception review process provided no practical oversight because post-hoc decisions by the RRBs cannot undo donor heart allocations that have already occurred. Furthermore, 174 people obtained transplants with status upgrades that were ultimately denied, bypassing thousands of other potential recipients due to inappropriately elevated priority. As a result, the retrospective review framework substantially undermines the system’s ability to allocate donor hearts fairly and with adequate oversight. In fact, this problem has worsened significantly over time, as evidenced in our results by the increasing absolute number of pre-RRB review heart transplants among exception candidates. OPTN policy documents^1^ report that centers that have transplanted candidates with denied exceptions are reported to the OPTN Heart Committee and potentially the Membership & Professional Standards Committee, a regulatory group that investigates noncompliance with OPTN policies and can enact disciplinary action, such as placing programs on probation.^13^ However, there are no publicly available data on whether these 174 cases were investigated and whether there were any consequences for the listing centers. With near-100% approval rates of all exception requests^3^ and unclear consequences for transplanting patients with unreviewed exceptions, the retrospective review framework appears to allow exceptions to serve as a pathway to heart transplantation that bypasses strict standard criteria with minimal oversight.

The reasons for retrospective review of exception applications are not well-known. One potential motivation for this system is to avoid high waitlist mortality among patients who need transplants urgently and may deteriorate during prospective review. However, patients with status exceptions generally have lower medical urgency compared with their counterparts who meet standard listing criteria.^8,9^ In fact, our study demonstrated very low rates of death or removal for deterioration before application receipt and final decisions were rendered in our sensitivity analysis. Nonetheless, policy initiatives to reduce the time to application receipt and review by RRBs may help further reduce this mortality.

As the OPTN deliberates on how to best review status exception requests,^14,15^ high rates of transplantation occurring before applications for status exceptions are reviewed should be avoided because such hearts are allocated without an adequate review of their waitlist priority. Centralization of exception review has already occurred in the pediatric heart allocation system with the National Heart Review Board’s implementation, which has been associated with reduced variation by center in status 1A exceptions.^16^ However, our results suggest that switching the system from retrospective to prospective review of exception requests is needed. Some inspiration can be taken from the liver allocation system. In May 2019, the OPTN established the National Liver Review Board, which centralized the prospective review of nonstandardized Model for End-Stage Liver Disease score exception requests and has been regularly updating comprehensive and transparent guidelines on applying for and reviewing applications.^17^ These significant changes have led not only to shorter times from application submission to decision but also significantly decreased approval rates without impacting waitlist mortality.^18^ Transplant centers must submit clinical narratives explaining why they are requesting status upgrades and how their candidates have similar medical urgency as others, achieving the requested priority through standard criteria.^1^ These narratives may help study how exceptions can be reviewed and granted, but such a task is difficult, as they are free-text paragraphs without any prespecified categories.

Instead, a potential first step in switching to prospective review of applications for exceptions in the heart allocation system is to use the US-CRS, a recently published continuous medical urgency score that is more accurate in rank-ordering candidates than the current 6-status system based on predicted 6-week waitlist mortality.^19^ US-CRS scores do not rely on hemodynamic measurements or choice of therapies and are heterogeneous among patients with status 1 and 2 exceptions,^20^ suggesting wide variation in true medical urgency among exception candidates. As a result, US-CRS scores could provide an efficient method of determining which patients applying for exceptions truly have high medical urgency when applications are reviewed upfront.

Overall, our results add to a growing body of work highlighting major shortcomings with status exceptions in heart allocation, including the increasing use of exceptions^4–7^ and the fact that exception candidates, in general, have significantly lower medical urgency than standard criteria candidates.^8,9^ Nonetheless, our study should not be construed as supporting the elimination of exception requests. Exceptions are meant to provide fairness and equity in access to transplant for candidates whose medical urgency and need for transplant are poorly represented by their priority set by the 6-status system. Such cases may include issues with access, like candidates who are highly sensitized and require a higher priority.^21,22^ However, the current framework for granting exceptions undermines principles of fairness and equity that are necessary for donor heart allocation.

## Limitations

We report several limitations. First, we were unable to calculate the time between application submission and actual review by the RRBs because the review date was not available in the SRTR data files. However, we demonstrated a high rate of transplantation even before the RRBs received the exception applications, and we performed a sensitivity analysis estimating a review date of 3 days after receipt of the applications.

Second, we made several assumptions when estimating the predicted statuses without exceptions at transplant for the 174 patients who were transplanted with exceptions that were ultimately denied. Our analysis assumes that transplant centers would not have recorded additional hemodynamic measurements or made other clinical changes if they had known immediately on application submission that the exception requests were denied. Because status listings for inotropic support and temporary mechanical circulatory support are highly dependent on the specific timing of recorded hemodynamic data, it is possible that the predicted statuses would be different if centers obtained more updated data, knowing earlier about application denial from the RRBs. Furthermore, there were several categorizations for standard listing for which the criteria could not be ascertained from the available data, which are detailed in the Supplemental Methods. For example, recurrent or sustained ventricular tachycardia/fibrillation is a standard listing category for status 2. However, the presence of an arrhythmia, unlike hemodynamic measurements and inotropic support, is not a standard variable collected within the status justification files unless transplant centers are specifically applying for status 2 with that category. As a result, it is possible that patients with exception denials could have potentially had a higher predicted status level than what was determined in our study if they actually met criteria for a categorization that was not possible to analyze.

## Conclusions

More than 25% of adult heart transplant candidates with submitted exception requests received heart transplants before their applications were even received by their respective RRBs, including those who have applied for exceptions that are ultimately denied. This raises significant concerns about the efficacy and fairness of retrospective review for the allocation of scarce donor hearts.

## Article Information

### Acknowledgments

The interpretation and reporting of these data are the responsibility of the authors and in no way should be seen as an official policy of or interpretation by the Scientific Registry of Transplant Recipients or the US government.

### Author Contributions

Drs Ahn, Parker, and Sasaki had full access to the data in the study and took responsibility for the integrity of the data and the accuracy of the data analysis. The concept and design were developed by Drs Ahn, Khush, Parker, and Sasaki. The acquisition, analysis, or interpretation of data were conducted by Drs Ahn, Nakayama, Parker, and Dr Sasaki and A. Attia and D. Eap. The drafting of the article was performed by Dr Ahn. The critical review of the article for important intellectual content was conducted by all authors. The statistical analysis was performed by Drs Ahn and Parker. The funding for the project was obtained by Dr Parker. Administrative, technical, and material support was provided by Drs Parker and Sasaki. The supervision of the project was overseen by Drs. Khush, Parker, and Sasaki.

### Disclosures

Dr Narang was a speaker for Boehringer Ingelheim and AstraZeneca and provided consulting services for BridgeBio. The other authors report no conflicts.

### Supplemental Material

Supplemental Methods

Tables S1–S4

Figures S1–S4

## Supplementary Material


