# Supplementary material for: High Rate of Transplantation Before Review of Status Exception Requests Among Adult Heart Transplant Candidates
Source: Circ Heart Fail. 2026 Jun 22;19(7):e013994. doi: 10.1161/CIRCHEARTFAILURE.125.013994 (PMC13288750; doi:10.1161/CIRCHEARTFAILURE.125.013994)
Supplement: Supplementary file 2 [file hhf-19-e013994-s002.pdf]

SUPPLEMENT MATERIAL

Table of Contents

Supplemental Methods

Supplemental Figures
- Figure S1
- Figure S2
- Figure S3
- Figure S4

Supplemental Methods

A. Information on the Justification File Setup

Information on whether transplant centers submitted applications for exceptions is available in the status justification files, which can be requested from the Scientific Registry of Transplant Recipients. The file of interest is the "JustFormHR," which is a parent data file that can be linked to other forms that contain information specific to the requested status.

Table S1

ID	JustID	Requested Status	Exception	FormAddDt	FormEffectiveDt	FormExpirationDt	FormReceiptDt	FormStatus
A	1	Status 3	0	2018-10-18	2018-10-18	2018-11-01	NA	Completed
A	2	Status 1	1	2018-10-31	2018-11-01	2018-11-08	2018-11-04	Approved
B	3	Status 2	1	2018-10-18	2018-10-18	2018-11-01	2018-10-23	Denied
B	4	Status 2	1	2018-10-23	2018-10-24	2018-11-07	2018-10-30	Denied
B	5	Status 3	0	2018-10-30	2018-10-31	2018-11-13	NA	Completed

Variable Descriptions:
ID: Patient ID
JustID: The unique index for every submitted justification form
RequestedStatus: The requested status for the patient
Exception: Denotes whether the justification form is an exception application or not.
FormEffectiveDt: The date when a justification form is both submitted and active
FormExpirationDt: The date when a form expires (how long a form remains active depends on the requested status upgrade)
FormReceiptDt: The date the assigned regional review board receives an application for an exception. This variable is "NA" for non-exception applications because they meet standard listing criteria and do not need to be reviewed.
FormStatus: The outcome of the justification form. Forms are simply "Completed" if they are for standard listing. These forms are not reviewed because patients must have met pre-specified criteria before submitting a justification form. For exception applications, the FormStatus is either "Approved" or "Denied" by the regional review boards. The data does not have the date when the board actually voted to approve or deny the forms. It only has the date the board actually received the forms for review, denoted by "FormReceiptDt."

Table S1 shows an example of how the justification files are coded. For patient A, a transplant center submitted a total of two justification files. The first one, labeled "1" in JustID, is for standard listing at status 3. The form was submitted and activated October 18, 2018 and expired November 1, 2018. Because it is a form for standard listing at status 3, there is no form receipt date, and it was simply just "Completed." However, the center submitted JustID "2" on the same day the JustID "1" expired. This was for status 1 with an exception. The form was submitted and activated on November 1, 2018 and was received by the regional review board 3 days later on November 4, 2018. The form was eventually approved, although we do not know when exactly. It is important to note that patient A had status 1 from November 1, 2018 to November 4, 2018 even though the application form had not even been received by the board yet.

For patient B, the center submitted an exception application (JustID “3”) for status 2 on October 18, 2018. It was received by the board on October 23, 2018. It was ultimately denied by the board. The center then submitted an appeal application on October 24, 2018, requesting the same status 2 with an exception. This appeal (JustID “4”) was then received by the regional review board on October 30, 2018, 6 days later. It was eventually denied. The transplant center did not submit any more exception applications for patient B and on November 8, 2018, they submitted JustID “5,” which was a request for status 3 at standard listing. The patient then had status 3 until the form expired on November 21, 2018. Because the review process is retrospective, it is important to note that even though patient B received two denials in a row, they had status 2 from October 18, 2018 (date of submission of JustID “3”) to at least November 7, 2018 (date of receipt of the appeal form JustID “4”).

B. Prediction of Status at Transplant without Exception

This section provides information on how we determined what status the 174 patients who received a transplant with an exception that was ultimately denied would have had if their exception was non-existent at the time of transplant.

In accordance with OPTN policy,[1] we defined high-dose inotropes as support by at least one of the following: ≥ 7.5 mcg/kg/min of dobutamine, ≥ 0.5 mcg/kg/min of milrinone, and ≥ 0.02 mcg/kg/min of epinephrine. We defined “multi-agent low dosage” as support by at least two of the following: ≥ 3 mcg/kg/min of dobutamine, ≥ 0.25 mcg/kg/min of milrinone, ≥ 0.01 mcg/kg/min of epinephrine, and ≥ 3 mcg/kg/min of dopamine. Either high-dose or multi-agent low-dose inotropes meets requirements for status 3 listing for inotropic support. To qualify for inotropic support for status 4, patients need to be supported by at least one of the following: ≥ 3 mcg/kg/min of dobutamine, ≥ 0.25 mcg/kg/min of milrinone, or ≥ 0.01 mcg/kg/min of epinephrine.

For patients who had a status achieved by meeting standard listing criteria just prior to obtaining an exception that was eventually denied, we simply carried forward the status that they had. This was the case for the majority of cases. For patients who were inactive before the exception application was submitted or the exception was the first status that they had ever submitted while on the waitlist, we predicted what status they would have had transplant with the following criteria:

Patients would have had status 1 in the following cases:

1. They were on VA-ECMO at listing
2. They were supported by a non-dischargeable BIVAD.
3. We were unable to determine whether patients could have life-threatening ventricular arrhythmia as this information is available only if transplant centers had submitted a status 1 request specifically for that reason.

Patients would have had status 2 in the following cases:

1. They were supported by an IABP at the time of initial listing and had the following recorded hemodynamic data in a 24-hour period within 7 days before submission of the application form

for the exception that was ultimately denied: PCWP > 15, SBP < 90, and cardiac index < 2 with inotropic support OR PCWP > 15, SBP < 90, and cardiac index < 1.8 without inotropic support.
2. They were supported by a percutaneous endovascular VAD and had the same hemodynamic data/timing of recorded measurements as listed above for IABPs.
3. They had a non-dischargeable LVAD, total artificial heart, BIVAD, right ventricular assist device, or ventricular assist device (for single ventricle patients) at any time before submission of the exception that was ultimately denied.
4. We could not determine whether patients had recurrent or sustained ventricular tachycardia/fibrillation or a device malfunction based on the data provided because this information is present only if transplant centers submitted status 2 requests for those reasons specifically. Whether a malfunction or ventricular tachycardia is ongoing is not standard information provided in the justification files.

Patients would have had status 3 in the following cases:
1. They had an invasive pulmonary artery catheter and were supported by high-dose or multi-agent low-dose inotropes meeting status 3 criteria (defined above) within 7 days before submission of the exception form that was denied.
2. We could not determine whether patients had a durable LVAD with the following complications (infection, pump thrombosis, right heart failure, aortic insufficiency) as this information is only available if transplant centers submitted forms specifically for those indications previously.

Patients would have had status 4 in the following cases:
1. They were supported by inotropes (no invasive hemodynamic monitoring necessary) meeting criteria for status 4, described above.
2. They were supported by a durable LVAD
3. They were a prior heart transplant recipient
4. They have a diagnosis code corresponding to restrictive cardiomyopathy, hypertrophic cardiomyopathy, or congenital heart disease

Patients would have had status 5 if they were listed for multi-organ transplantation.

Patients would have had status 6 if none of the cases above applied for them.

C. *Match Run Analysis*

We performed a match run analysis using complete match run data provided by the SRTR to determine how many potential transplant recipients were skipped by the patients who obtained transplants with active exceptions that were ultimately denied. To perform this analysis, one must understand how the donor hearts are actually allocated. They are allocated based on four categories: status, distance between the donor hospital and the transplanting hospital, blood type, and donor age. Donor hearts are allocated through group classifications that are detailed within OPTN policy.[1] Here is a screenshot of a section of a table taken directly from OPTN policy documents[1]:

Table S2

Classification	Candidates that are within the	And registered at a transplant hospital that is at or within this distance from the donor hospital
**1**	Adult status 1 or pediatric status 1A and primary blood type match with the donor	500NM
**2**	Adult status 1 or pediatric status 1A and secondary blood type match with the donor	500NM
**3**	Adult status 2 and primary blood type match with the donor	500NM
**4**	Adult status 2 and secondary blood type match with the donor	500NM

As shown in Table S2, for allocation of a deceased donor heart from a donor at least 18 years old, it is first offered to transplant centers with waitlisted patients within classification 1 (must have adult status 1 or pediatric status 1A, have primary blood type match with the donor, and be within 500 nautical miles of the donor hospital). There can be a significant number of patients within each classification. Patients are rank ordered within each classification based on waiting time. If no transplant center with a patient within classification 1 accepted the donor, then the donor heart gets offered to patients in classification 2. This process continues until a transplant center accepts the heart. There are 68 classifications for donors 18 years and older and 104 classifications for donors less than 18 years of age. An example of the analysis we performed is provided below:

Table S3

MATCH_ID	DONOR_ID	PX_ID	SEQUENCE_NUM	CLASSIFICATION
1	1	A	1	1
1	***1***	***B***	***2***	***1***
1	1	C	3	1
1	1	D	4	2
1	1	E	5	2
1	1	F	6	2
1	1	G	7	2
1	1	H	8	2
1	1	I	9	2
1	1	J	10	2
1	1	K	11	2
1	1	L	12	2
1	1	M	13	3
1	1	N	14	3

Variable Descriptions:

MATCH_ID: ID of the unique match run performed for a single donor heart that is available
DONOR_ID: Unique ID indexed to a donor

PX_ID: ID indexed to a potential transplant recipient
SEQUENCE_NUM: Denotes the order of patients in which a donor heart is offered
CLASSIFICATION: The grouping of patients to whom a donor heart is offered.

In Table S3, we have a patient B (bolded and italicized) who was sequence number 2 through an exception for status 1. The center at which patient A was listed did not accept the donor heart, so the organ was then offered to the center where patient B was. After accepting the organ, patient B received a transplant. The exception that this patient had was denied, and they should have been status 2. Based on Table S2, this patient should have been in classification 3. As mentioned in the main manuscript, we simply assumed that patients would be at the top of their classification.

Table S4

MATCH_ID	DONOR_ID	PX_ID	SEQUENCE_NUM	CLASSIFICATION
1	1	A	1	1
1	1	B	2	1
1	1	C	3	1
1	1	D	4	2
1	1	E	5	2
1	1	F	6	2
1	1	G	7	2
1	1	H	8	2
1	1	I	9	2
1	1	J	10	2
1	1	K	11	2
1	1	L	12	2
1	1	B	13	3
1	1	M	14	3
1	1	N	15	3

Thus, as shown in Table S4, patient B in reality should have been sequence number 13 at the top of the 3rd classification group. In this hypothetical scenario, by getting transplanted with a status 1 exception that was ultimately denied, patient B skipped 10 potential transplant recipients.

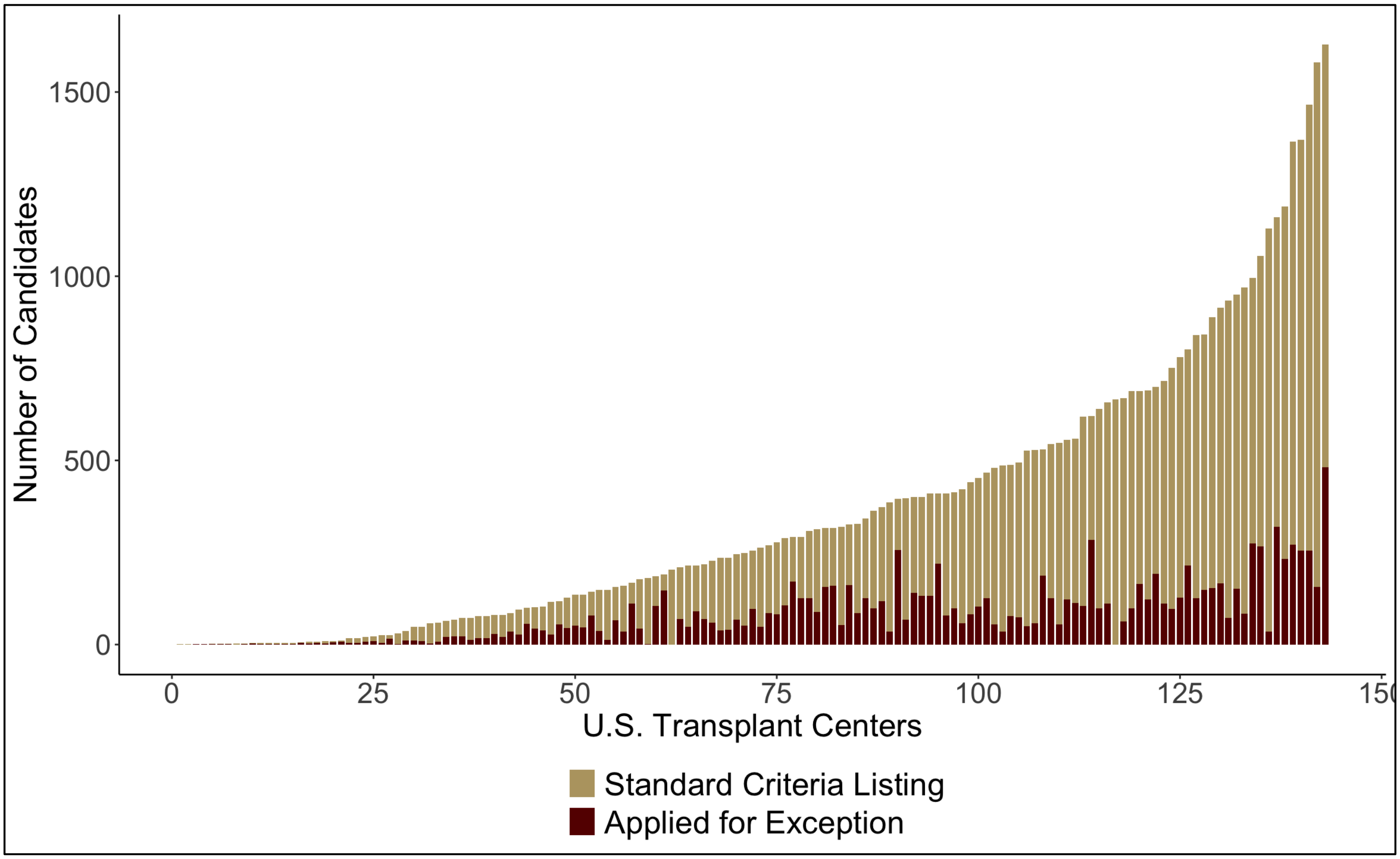

Figure S1: Distribution of proportion of candidates with submitted exception applications, stratified by US transplant center.

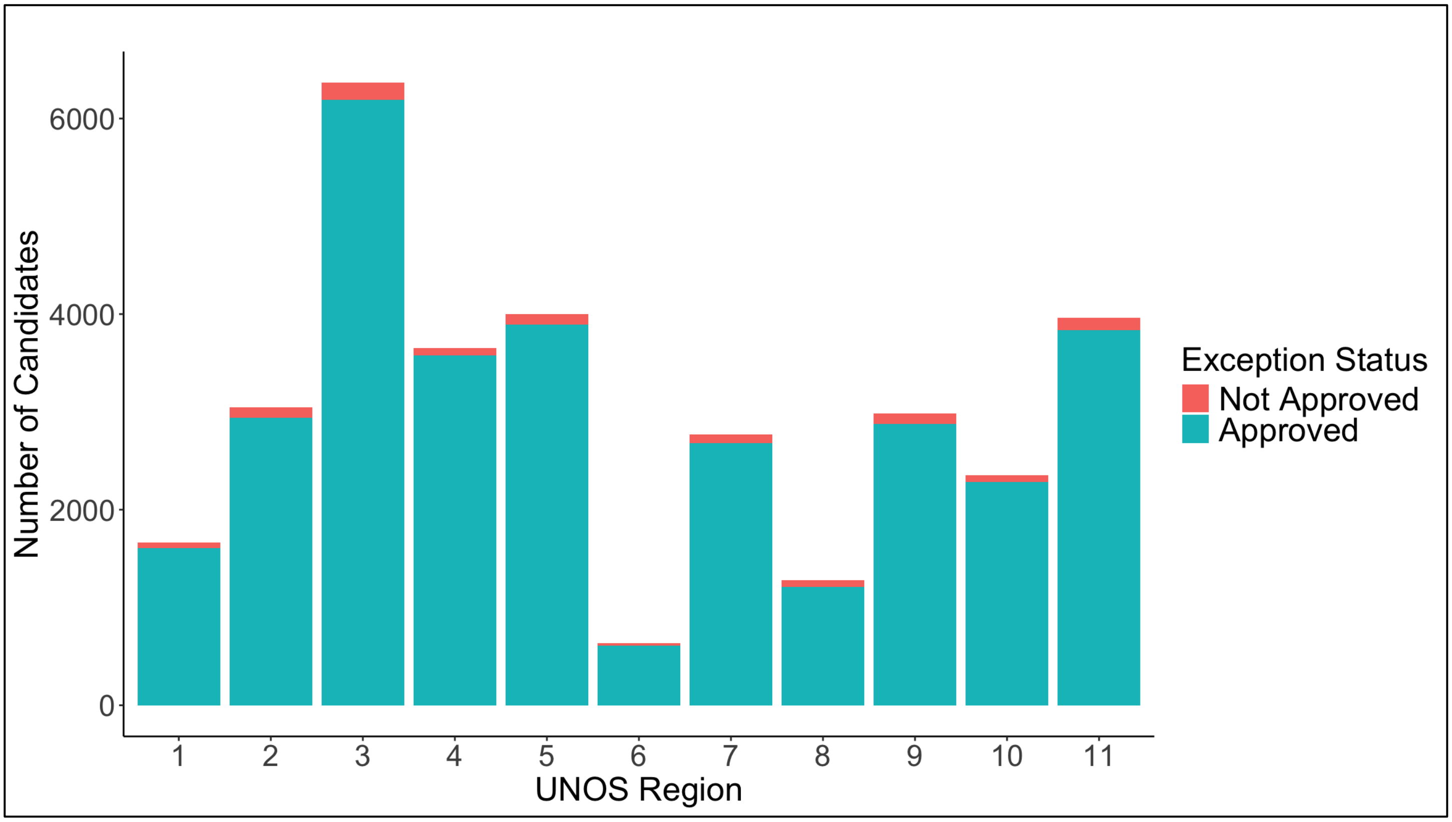

Figure S2: Absolute number of adult heart transplant candidates with approved exception applications, stratified by UNOS region

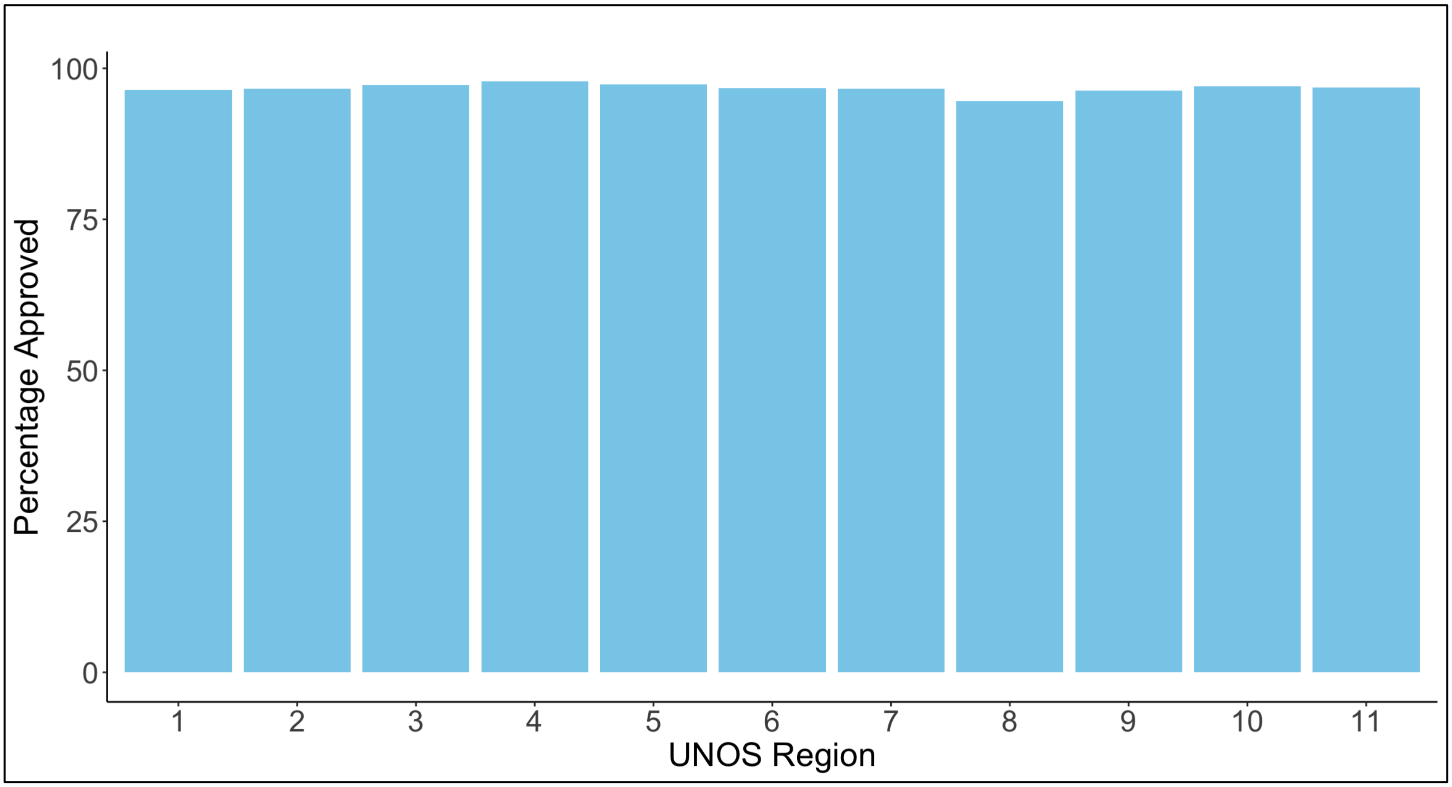

Figure S3: Proportion of adult heart transplant candidates with approved exception applications, stratified by UNOS region

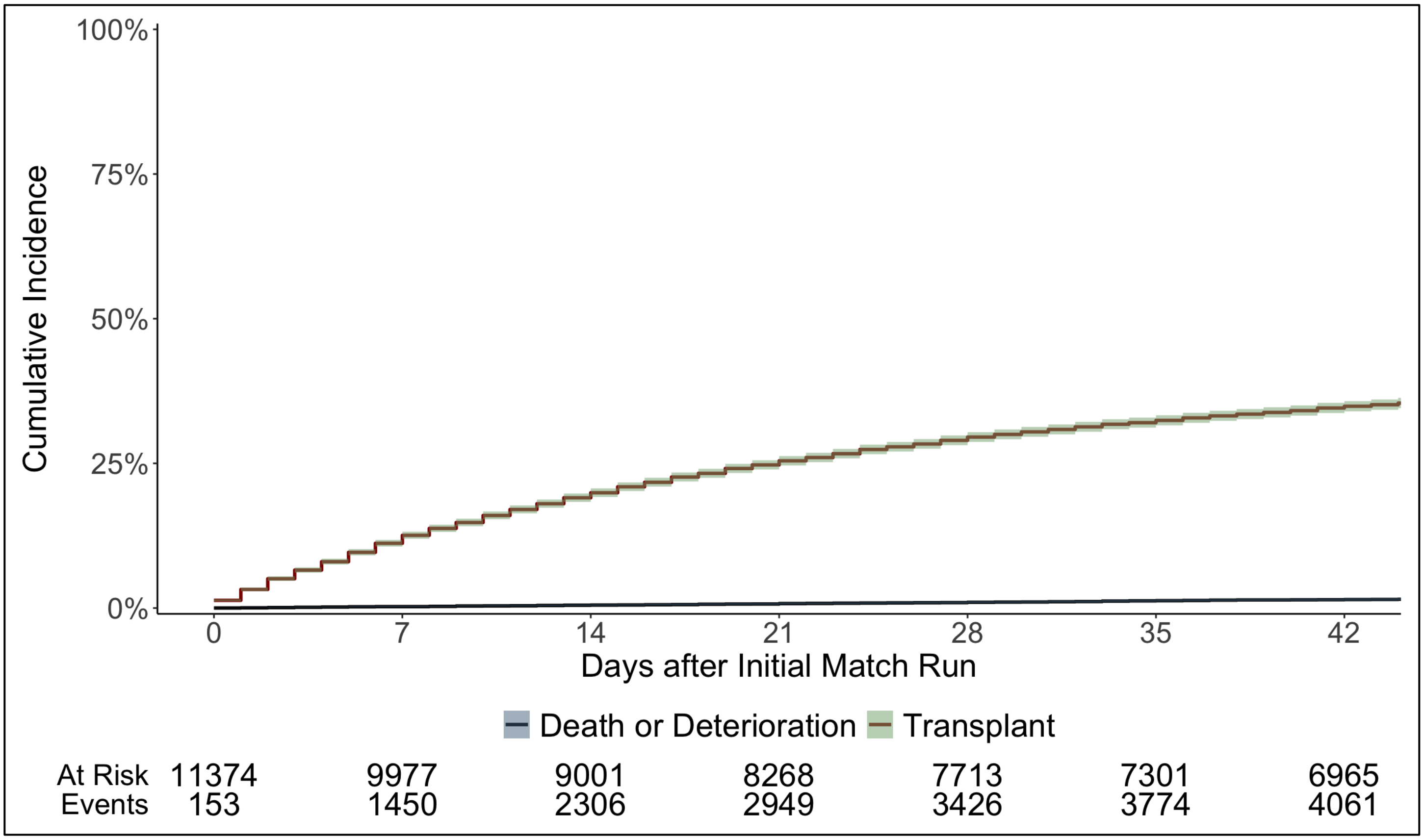

Figure S4: Cumulative incidence within 6 weeks of death or removal for clinical deterioration, treating transplantation as a competing event, of potential transplant recipients bypassed by candidates who obtained heart transplants with status exceptions that were eventually denied by the regional review boards.
